# Supplementary material for: KAT3-dependent acetylation of cell type-specific genes maintains neuronal identity in the adult mouse brain
Source: Nat Commun. 2020 May 22;11:2588. doi: 10.1038/s41467-020-16246-0 (PMC7244750; doi:10.1038/s41467-020-16246-0)
Supplement: Supplementary file 1 — Supplementary Information [file 41467_2020_16246_MOESM1_ESM.pdf]

Supplementary Figures and Tables

# KAT3-dependent acetylation of cell type-specific genes maintains neuronal identity in the adult mouse brain

Lipinski et al.

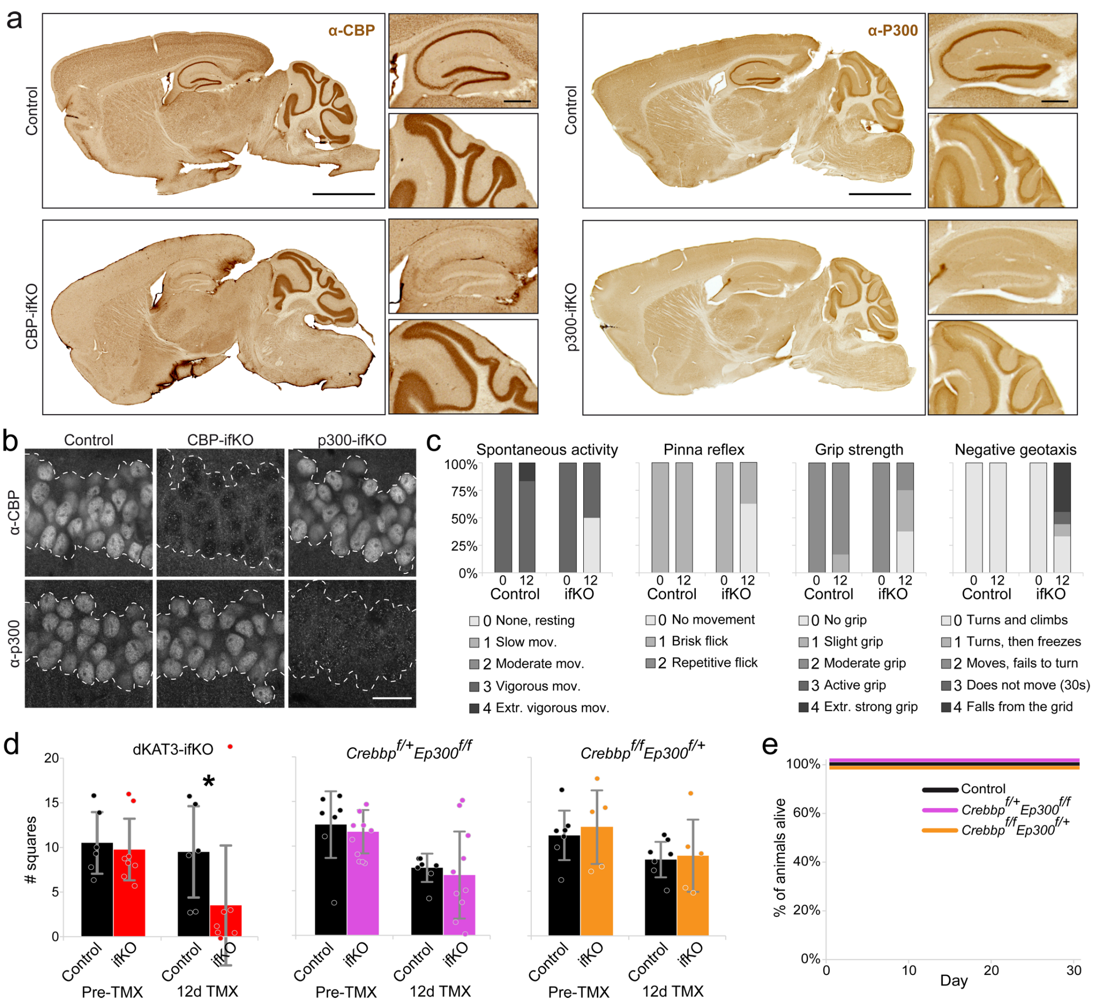


**Supplementary Figure 1 related to Figure 1. Conditional ablation of KAT3 genes in forebrain excitatory neurons. a.** DAB immunostaining showing CBP/p300 loss in forebrain neurons Scale: 5 mm. Note that the expression of these proteins in the cerebellum is not altered (insets; scale: 1 mm). IHC α-CBP: n = 2, IHC α-p300: n = 3, each repeated twice. **b.** IHC against CBP and p300 in the CA1 region of control, CBP-ifKO and p300-ifKO mice. Dashed line contour indicates the position of the nuclei based on the DAPI signal. The elimination of both proteins can be observed in pyramidal and granule neurons (not shown) in the hippocampus. Experiment replicated twice with different sets of mice. Scale: 10 μm. **c.** Score of dKAT3-ifKO and control littermates in several significantly affected SHIRPA categories. The animals were scored twice: right before starting the TMX treatment ("0") and 12 days after the first TMX administration ("12"; Control, n = 6; dKAT3-ifKO, n = 8). Each category is scored according to a specific scale. The details regarding the scoring rules can be found in the Methods and Supplementary Table 1. **d.** Locomotor activity in the open field as measured by the number of entered squares. Values for control mice are shown in black, CaMKIIα-CreERT2::Crebbp$^{f/f}$::Ep300$^{f/f}$ in red, *CaMKIIα-CreERT2::Crebbp$^{f/+}$::Ep300$^{f/f}$* in magenta, and *CaMKIIα-CreERT2::Crebbp$^{f/f}$::Ep300$^{f/+}$* in orange. The left panels show the results before TMX treatment and the right panels,12 days after the first TMX administration. Two-sided Mann-Whitney U test: *: p-val = 0,040. Data are presented as mean ± SEM. **e.** Survival curve during the first month after TMX for control (n = 15), *CaMKIIα-CreERT2::Crebbp$^{f/+}$::Ep300$^{f/f}$* (n = 10) *and CaMKIIα-CreERT2::Crebbp$^{f/f}$::Ep300$^{f/+}$* (n = 6). Source data for graphs in panels c, d and e are provided as a Source Data file.

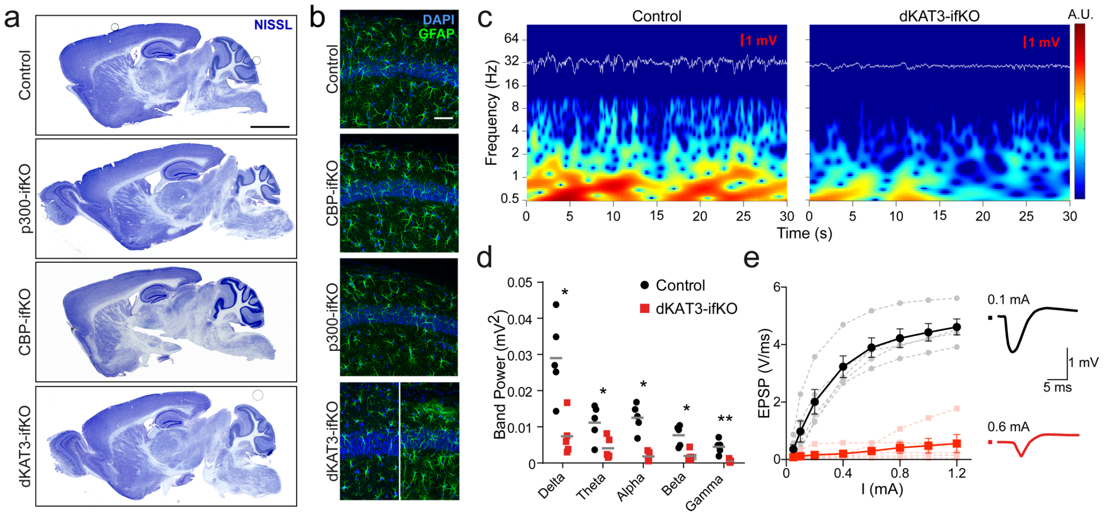


**Supplementary Figure 2 related to Figure 1. Reduction in the number of synapses and neuronal activity in dKAT3-ifKOs. a.** Nissl staining showing normal gross anatomy and histology 1 month after single and combined CBP/p300 ablation. Scale: 5 mm. The experiment was repeated twice. **b.** Immunostaining against GFAP shows no gliosis in the hippocampus of CBP-ifKOs, p300-ifKOs and in most dKAT3-ifKOs. Although we observed moderate active gliosis in a subset of dKAT3-ifKOs (less than 10%), this was not a general feature of dKAT3-ifKO brains. Instead, gliosis may result from the occasional episodes of epilepsy observed in some dKAT3-ifKO mice in the first days after gene ablation. Scale: 50 µm. The experiment was repeated twice. **c.** Wavelet spectra of dentate gyrus Local Field Potentials (LFPs) from control (left) and mutant (right) mice. LFP signal from the same channel is presented as a white line in the upper part of each panel. A.U. – arbitrary unit. **d.** Dentate gyrus LFPs band-power in the delta (0-4 Hz), theta (4–8 Hz), alpha (8–13 Hz), beta (13–30 Hz), and gamma (30–125 Hz) frequency bands. Two-sided Mann-Whitney U test: *: $p\text{-val}_{delta}$ = 0.012, $p\text{-val}_{theta}$ = 0.012, $p\text{-val}_{alpha}$ = 0.012, $p\text{-val}_{beta}$ = 0.012: **: $p\text{-val}_{gamma}$ = 0.008. **e.** Left: Quantification of EPSP slope of the stimulus-response curve recorded in dentate gyrus after perforant pathway stimulation for control (black) and mutant (red) mice. Right: representative EPSPs evoked potentials. Note the six-time larger stimulation intensity applied to dKAT3-ifKOs. In panels **d** and **e**, the data are presented as mean values ± SEM. For Control and dKAT3-ifKO, n = 5. Source data for graphs in panels d and e are provided as a Source Data file.

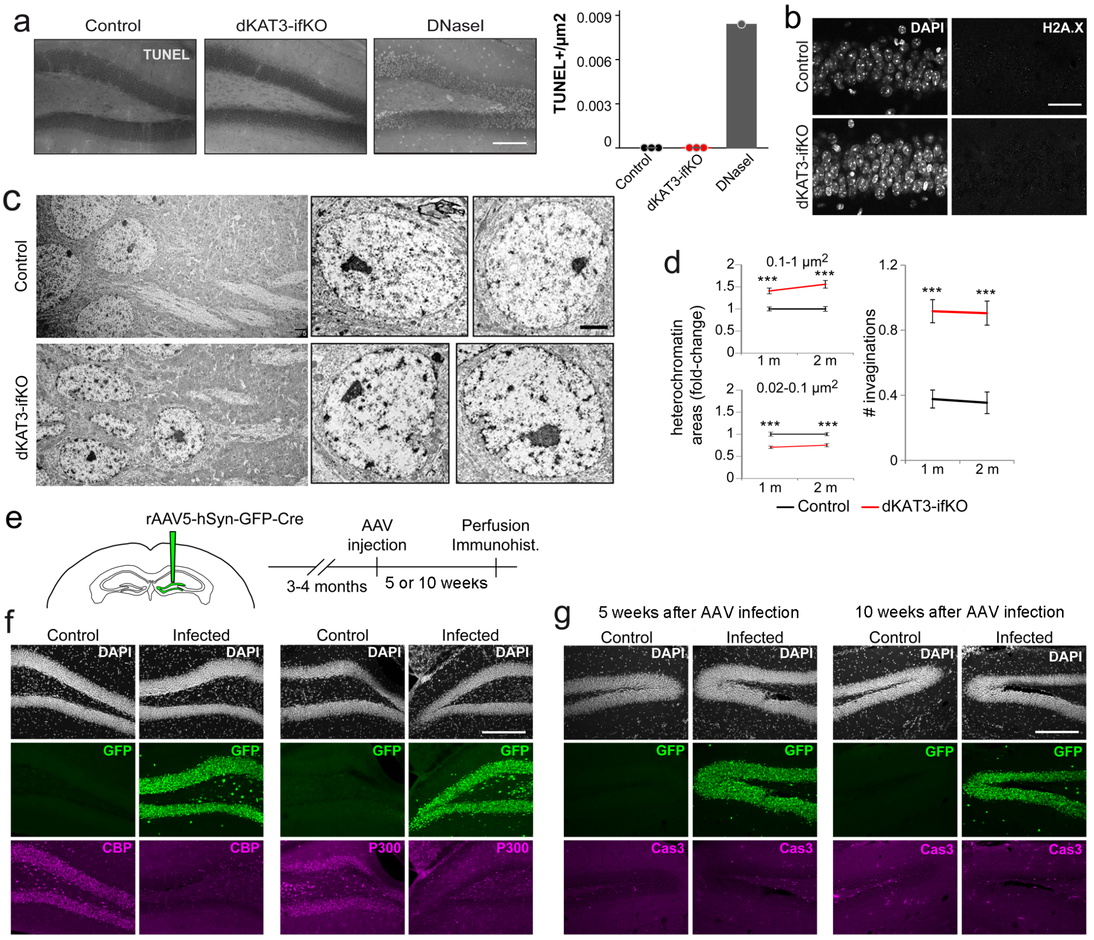


**Supplementary Figure 3 related to Figure 1. Combined ablation of CBP and p300 does not cause neuronal loss. a.** TUNEL staining images (top) and quantification (below) show no cell death either in the brain of mice from either genotype. A section treated with DNase I was used as a positive control. Scale: 200 µm. Control and dKAT3-ifKO, n = 3, DNaseI positive control, n = 1. **b.** Immunostaining against phosphorylated (pS139) histone H2A.X gamma, a marker of DNA damage related to cell death. Scale: 30 µm. **c.** Electron microscopy (EM) images of the *strata pyramidale* and *radiatum* (left; scale: 5 µm.) and representative CA1 nuclei (right; scale: 2 µm) in a control and a dKAT3-ifKO mouse 1 month after TMX. **d.** Quantification of nuclear features in EM images 1 and 2 months after TMX. Left: number of large (0.1 - 1 $\mu m^2$, top) and small (0.02 - 0.1 $\mu m^2$, bottom) electrodense granules per nucleus. Right: number of nuclear envelope invaginations per nucleus. Statistic: t-test. For both Control and dKAT3-ifKO, the values are averages of 30 areas from 3 mice per condition and the error bars represent SEM. Two-tailed t-test: ***: p-val < 0.0001. **e.** The upper scheme describes the AAV-Cre-GFP stereotaxic injection experiment. AAV-Cre-GFP virus was injected unilaterally into the DG of adult *Crebbp$^{f/f}$::Ep300$^{f/f}$* mice. **f.** Immunostainings showing the loss of both CBP and p300 in AAV-cre transduced Crebbp$^{f/f}$::Ep300$^{f/f}$ mice. The virus was injected unilaterally into the DG of adult *Crebbp$^{f/f}$::Ep300$^{f/f}$* mice and the mice were perfused 1 month later. Scale: 200 µm. **g.** Immunohistochemistry for Cleaved Cas3 in AAV-cre-transduced mice. Mice were perfused five (left) or ten (right) weeks after viral infection. The experiment was repeated three times. Scale: 200 µm. Source data for graphs in panels a and d are provided as a Source Data file.

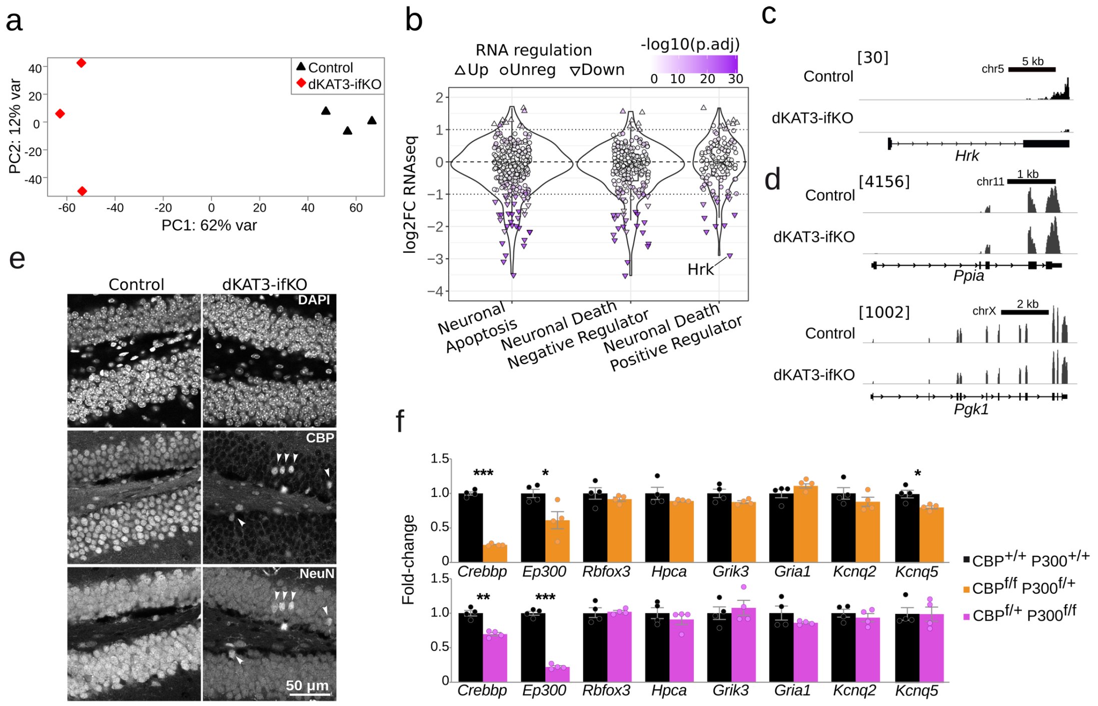


**Supplementary Figure 4 related to Figure 2. Hippocampal neurons lacking KAT3 fail to express neuronal-specific genes. a.** Principal component analysis plot of the RNA-seq experiment comparing hippocampal RNA of dKAT3-ifKO and control littermates. **b.** The genesets related with apoptosis and cell death (according to Gene Ontology) are not differentially expressed in dKAT3-ifKOs. Each dot represents a single gene. Red: upregulated genes; blue: downregulated genes; grey: no change. Number of genes per boxplot: neuron_apoptosis = 265, neuron_death_reg_negative = 212, neuron_death_reg_positive =1,116. The change of the most severely downregulated positive regulator of neuronal death, *Harakiri* (*Hrk*), is labeled. **c.** RNA-seq profile for *Hrk* in dKAT3-ifKOs and control littermates. **d.** Examples of RNA-seq profiles for two housekeeping genes: *Ppia* (peptidylprolyl isomerase A) and *Pgk1* (phosphoglycerate kinase 1). **e.** Immunostaining against CBP and NeuN focusing in a rare field of the dentate gyrus containing a few cells (possibly interneurons) in which there was no recombination and NeuN expression was maintained (arrowheads) The experiment was repeated twice with different set of mice. Scale: 50 µm. **f.** RT-PCR analysis in the hippocampus of CaMKIIα-CreERT2::*Crebbp*$^{f/+}$::*Ep300*$^{f/f}$ (n = 4), CaMKIIα-CreERT2::*Crebbp*$^{f/f}$::*Ep300*$^{f/+}$ (n = 4) and Control (n = 4) littermates. The expression of target genes was decreased more than 2-fold in the hippocampi of dKAT3-ifKOs (**Supplementary Data 1**). Notice that only *Kcnq5* shows a slight downregulation in CaMKIIα-CreERT2::*Crebbp*$^{f/f}$::*Ep300*$^{f/+}$ tissue Data are presented as mean values ± SEM. Two-tailed t-test: ***: $p < 0.0001$, **: $p < 0.01$; *: $p < 0.05$. Source data for graphs in panels b and f are provided as a Source Data file.

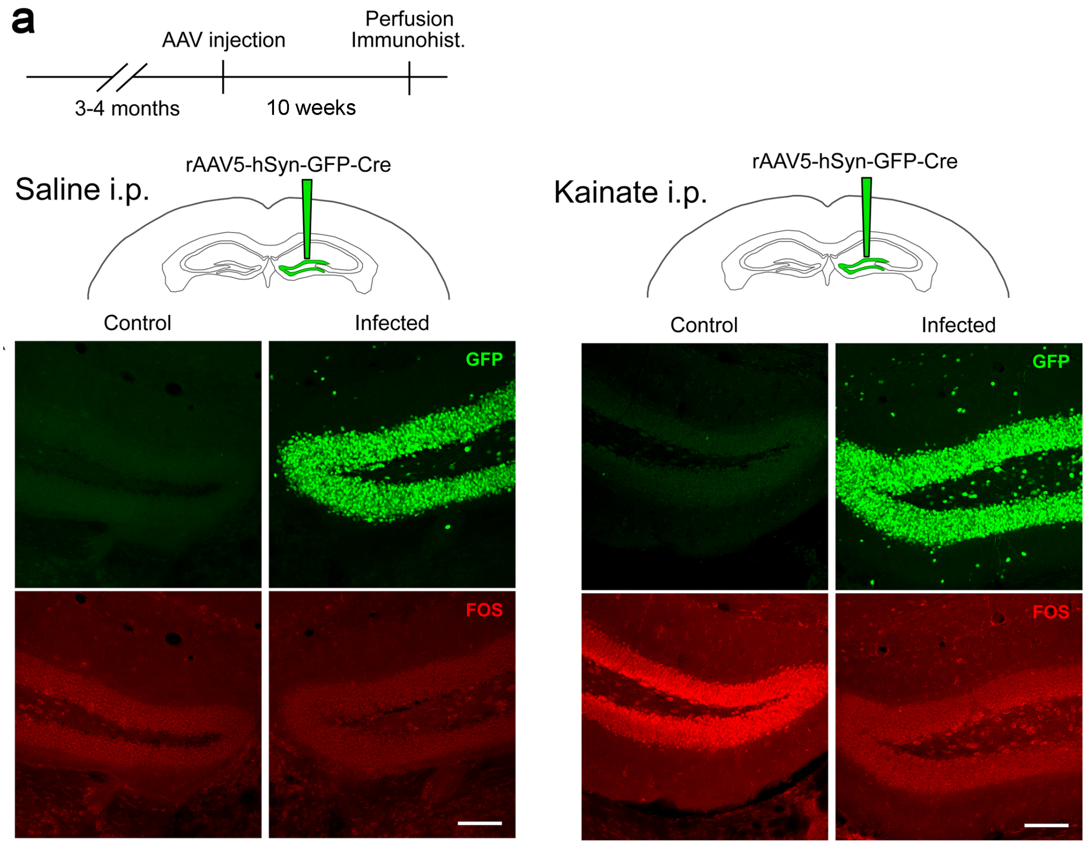


**Supplementary Figure 5 related to Figure 2. Cell autonomous loss of neuronal responsiveness after KAT3s ablation.** Adult *Crebbp*$^{f/f}$*::Ep300*$^{f/f}$ mice with monolateral AAV-Cre-GFP infection in the dentate gyrus. After ten weeks, the mice were injected intraperitoneally (i.p.) with saline or kainic acid and perfused one hour later during the *status epilepticus*. Immunostaining against Fos demonstrates that only the neurons in the non-injected site robustly respond to kainic acid. The experiment was repeated twice with different set of animals. Scales: 200 µm.

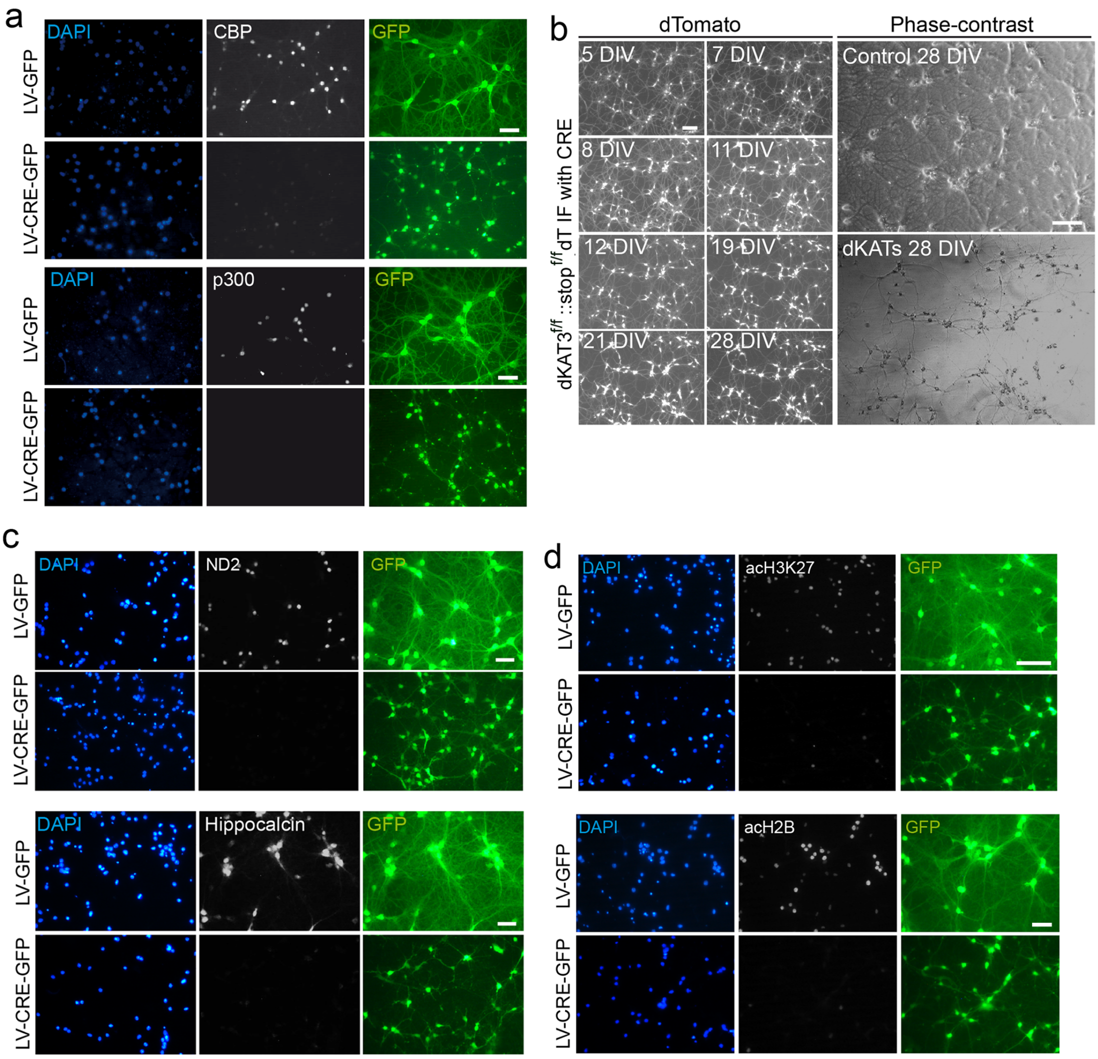


**Supplementary Figure 6 related to Figure 2. KAT3s safeguard the identity of cultured neurons. a.** Immunocytochemistry images showing a robust elimination of CBP and p300 in the GFP-positive neurons of the dKAT3-cKO cultures. Scale bar: 50 µm. **b.** Maintenance of neuronal viability in dKAT3$^{f/f}$::stop$^{f/f}$-tdTomato (tdT) hippocampal neurons infected with LV-CRE up to 27 days post infection (n = 3**)**. Scale bar: 100 µm. **c.** Immunostainings against NeuroD2 and hippocalcin reveal the reduced expression of these neuronal markers in dKAT3$^{f/f}$ hippocampal neurons infected with LV-Cre. Scale bar: 50 µm. **d.** Immunostaining against H3K27ac and H2Bac in PNC from dKAT3$^{f/f}$ hippocampi infected with LV-Cre or the control LV-GFP. Scale bar: 50 µm. The results presented in figures a, c and d were observed in six independent hippocampal cultures.

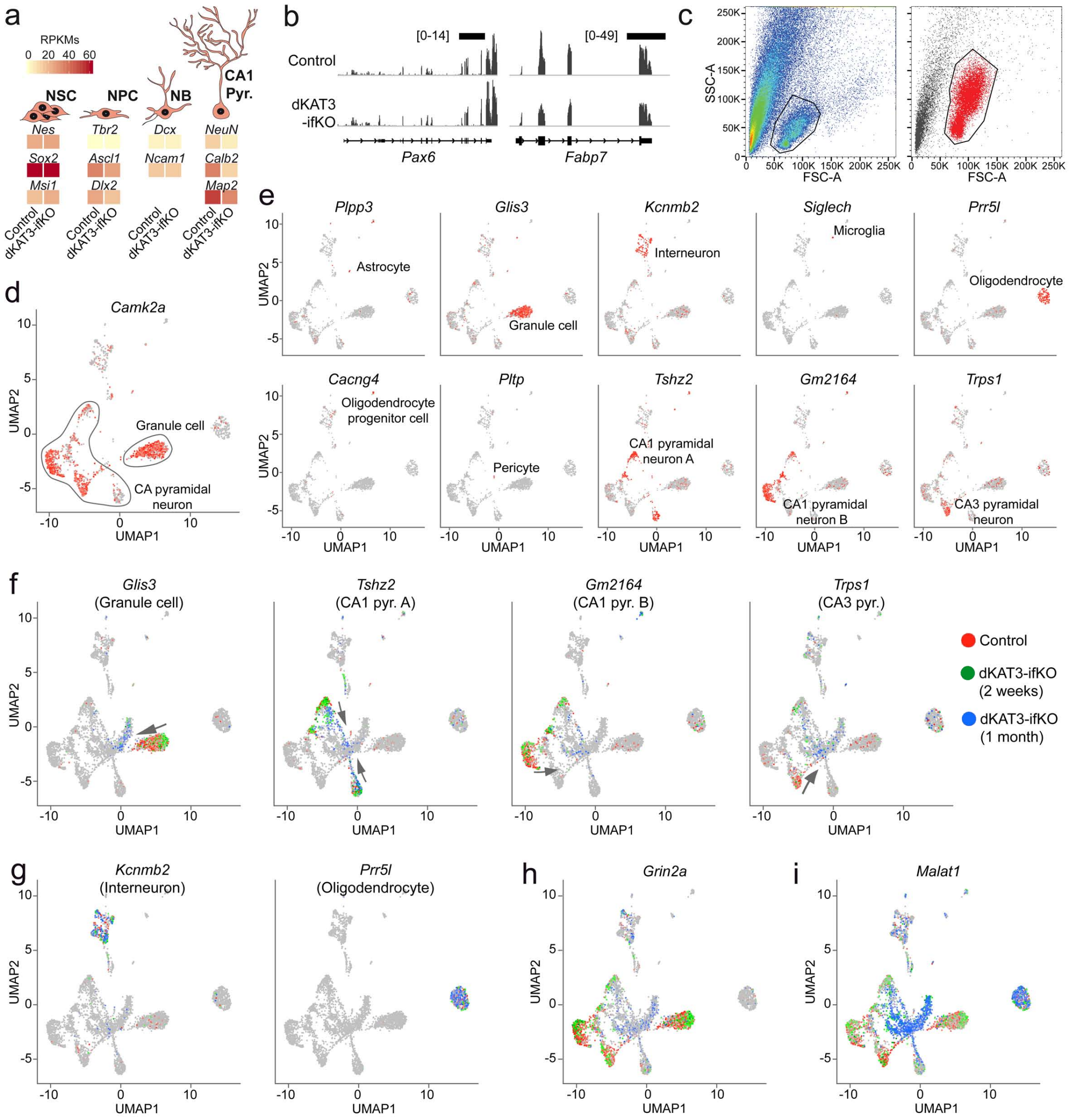

**Supplementary Figure 7 related to Figure 3. Loss of neuronal identity examined by single-nucleus RNA-seq. a.** Heat boxes showing the change in expression of genes involved in neurogenesis [42] and the maintenance of neuronal identity [43] in our mRNA-seq data. NSC: Neuronal stem cells; NPC: Neuroprogenitor cells; NB: Neuroblast; CA1 Pyr: CA1 Pyramidal Neurons. **b.** mRNA-seq track for representative genes enriched in NPCs. Scale: 5 kb for *Pax6* and 1 kb for *Fabp7*. **c.** Left: Flow cytometry graphs showing the gate used to isolate singlet nuclei. Right: Sorting accuracy was confirmed by DAPI staining and re-sorting the nuclei. Singlet nuclei positive for DAPI are labelled in red. **d.** UMAP plot showing levels of *Camk2a* transcripts in the control dataset. Note that *Camk2a* expression is restricted to major excitatory neuronal populations such as CA1 and CA3 pyramidal and dentate gyrus granule cells (contours). **e.** Expression of selected subpopulation-specific gene markers in the single-nucleus RNA-seq dataset of control mice. **f-g.** UMAP plots of integrated datasets from the three time points showing the expression level of selected subpopulation-specific genes. The position of the cells expressing gene markers for granule cells (*Glis3*) and CA1 (*Gm2164, Tshz2*) and CA3 (*Trps1*) neurons that are still detected in dKAT3-ifKOs, shifts towards the novel central cluster (**f**). In contrast, the position of cells expressing gene markers for spared populations, such as *Kcnmb2* (interneurons) and *Prr5l* (oligodendrocytes), remain stable (**g**), **h-i.** UMAP plots of integrated datasets showing the disappearance of cells expressing excitatory neuron-specific genes (e.g., *Grin2a*; **h**) and the appearance of a new cluster characterized by the upregulation of non-cell-specific genes (e.g., *Malat1*; **i**).

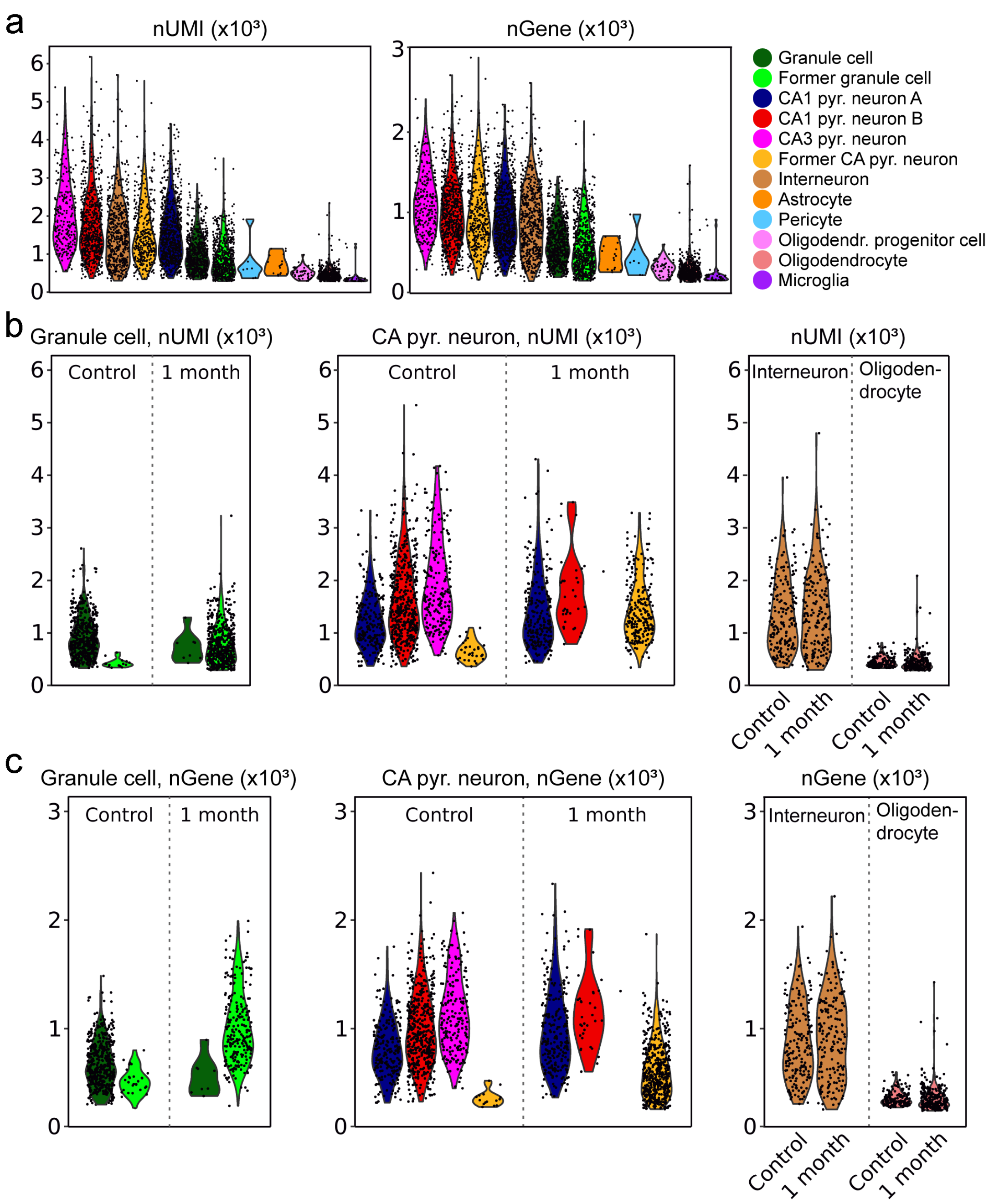


**Supplementary Figure 8 related to Figure 3. Transcript diversity and abundance in dKAT3-KO cells. a.** Violin plots showing total UMI (nUMI) per cell (left) and total number of detected genes (nGene) per cell (right) for the different subpopulations identified in the mouse hippocampus. In each plot, cell populations are ranked by their respective mean value. Cell populations are color coded as indicated in the legend. **b.** Violin plots showing total UMI (nUMI) for granule cell and former granule cell (left), CA pyramidal neurons and former CA pyramidal neurons (middle), and interneurons and oligodendrocytes (right), split by condition (i.e., time upon dKAT3s ablation, the sample for 2 weeks show an intermediate result as expected). **c.** As **panel b** but showing total number of detected genes (nGene) per cell cluster. Source data are provided as a Source Data file.

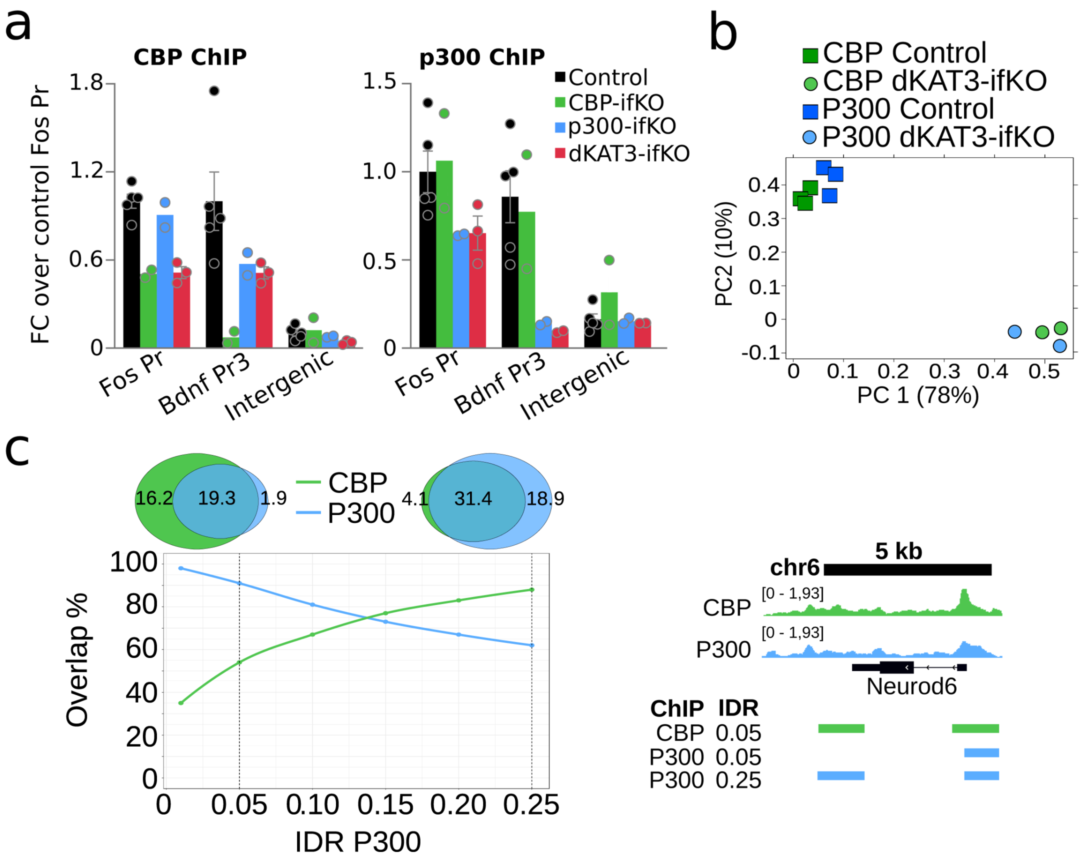


**Supplementary Figure 9 related to Figure 4. CBP and p300 bind to the same places throughout the mouse genome. a.** Specificity of the CBP and p300 antibodies in ChIP assays. The *Fos* and *Bdnf* promoters (Pr) are occupied by both KAT3 proteins, while an intergenic location (negative control) is not. Control n = 4, CBP-ifKO n = 2, p300-ifKO n = 2, dKAT3-ifKO n=3 (CBP ChIP) or n = 2 (p300 ChIP). **b.** Principal component analysis (PCA) of CBP and p300 ChIP-seqs from control and dKAT3-ifKO. CBP and p300 ChIP-seq samples cluster perfectly depending on the genetic background, indicating they are nearly identical. Genotype explains 78% of the variance. **c.** Left: Overlap of KAT3 peaks CBP and p300 depends on the threshold for Independent Discovery Rate (IDR) used. Here, we maintained a constant IDR of 0.05 for CBP ChIP-seq and calculated the overlap with the p300 ChIP-seq peaks obtained by increasing the IDR in steps of 0.05. Numbers in Venn plots indicate peaks in thousands, lines in the graph indicate % of overlap. At IDR 0.05, most of the p300 peaks (~90%) colocalizes with CBP. With and IDR for p300 of 0.25, most CBP peaks (~90%) co-localizes with p300. Right: The upper profiles show two examples of CBP peaks that are detected or not in p300 profiles, depending on the IDR threshold used. The enrichment for p300 signal over background in clearly observed in both regions. Source data for graphs in panel a are provided as a Source Data file.

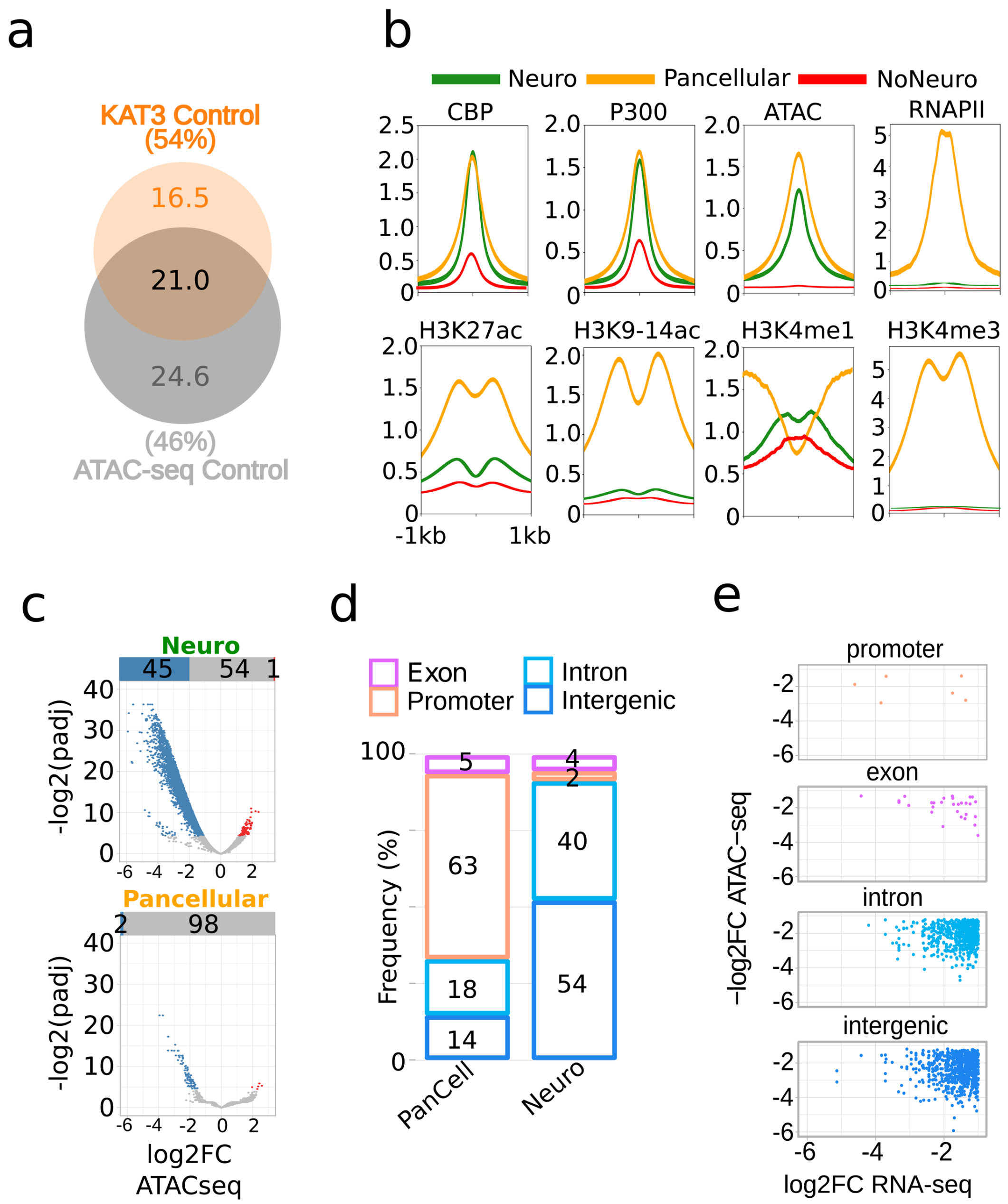


**Supplementary Figure 10 related to Figure 4. Classification of KAT3 peaks according to cell type and gene features. a.** Overlap between ATAC and KAT3 (CBP + P300) peaks. Numbers of regions detected in each screen in thousands. **b.** Metaplots showing the signal for proteins and histone post-translational modifications associated with promoters (H3K4me3, H3K9-14ac and RNAPII) and enhancers (H3K27ac and H3K4me1, as well as CBP and p300 binding), and chromatin accessibility profiles. Data is provided for the sets of neuronal (green), non-neuronal (red) and pancellular (orange) KAT3 peaks. **c.** Distribution of DARs in neuronal and pancellular KAT3 peaks in dKAT3-ifKOs. **d.** Changes in ATAC-seq signal at neuronal and pancellular peaks. **e.** Relationship between loss of accessibility and gene expression for all peaks in dKAT3-ifKO genetic background divided by genomic feature. Both types of change occur almost exclusively in intergenic and intronic regions.

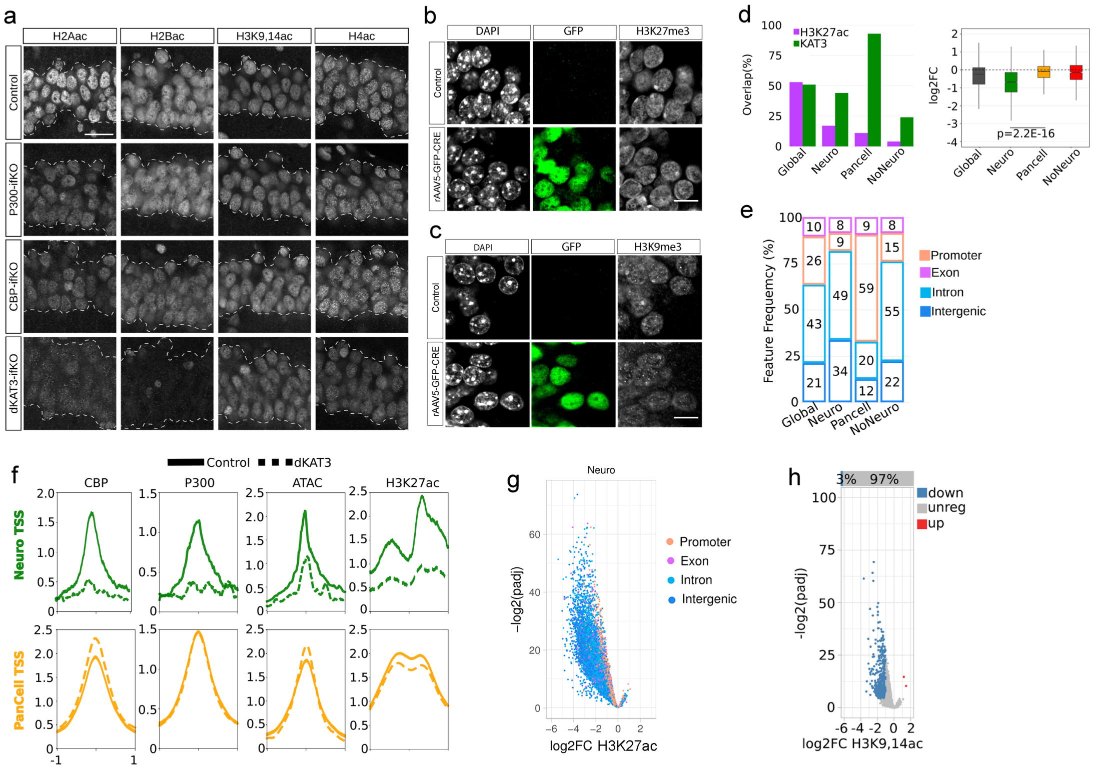


**Supplementary Figure 11 related to Figure 5. H3K27ac hypoacetylation correlates with gene downregulation**. **a**. Immunostaining against different acetylated lysine residues in CA1 pyramidal neurons of control mice and the different ifKOs. Scale: 10 µm. **b-c**. Immunostaining for H3K27me3 (b) and H3K9me3 (c) in the dentate gyrus of adult *Crebbp*$^{f/f}$::*Ep300*$^{f/f}$ mice 2 months after monolateral AAV-Cre-GFP infection in the dentate gyrus. Scale: 10 µm. **d.** Left: Percentage of overlap between H3K27ac and KAT3 peaks in the chromatin of control mice. We present the values for all peaks (Global) and the cell type-specific subsets. Right: Transcript level changes in the genes associated with the four peak subsets defined in the left graph. The number of genes represented in each box are: Global = 12,002, Neuro = 2,740, Pancell = 3,093, NoNeuro = 959. Whisker lengths are 1.5 the interquartile range of the box. Gene sets associated with Neuro and Pancell peaks are compared using a Wilcoxon test, p-value = 2.2e-16. **e.** Genomic distribution of H3K27ac peaks in control mice categorized by genomic feature (global) and overlap with neuronal, pancellular and non-neuronal KAT3 peaks. **f.** Metaplots of ATAC-seq, KAT3 and H3K27ac ChIP-seq signals in neuronal and pancellular promoters in controls and dKAT3-ifKOs. **g.** Volcano plots presenting fold change and significance values for neuron-specific peaks (as in the middle panel of **Fig. 5b**). Dots are colored according to their location in promoters, or intragenic and intergenic regions. **h.** Volcano plot presenting the changes of H3K9,14ac in dKAT3-ifKOs hippocampal chromatin. Source data for graph in panel d are provided as a Source Data file.

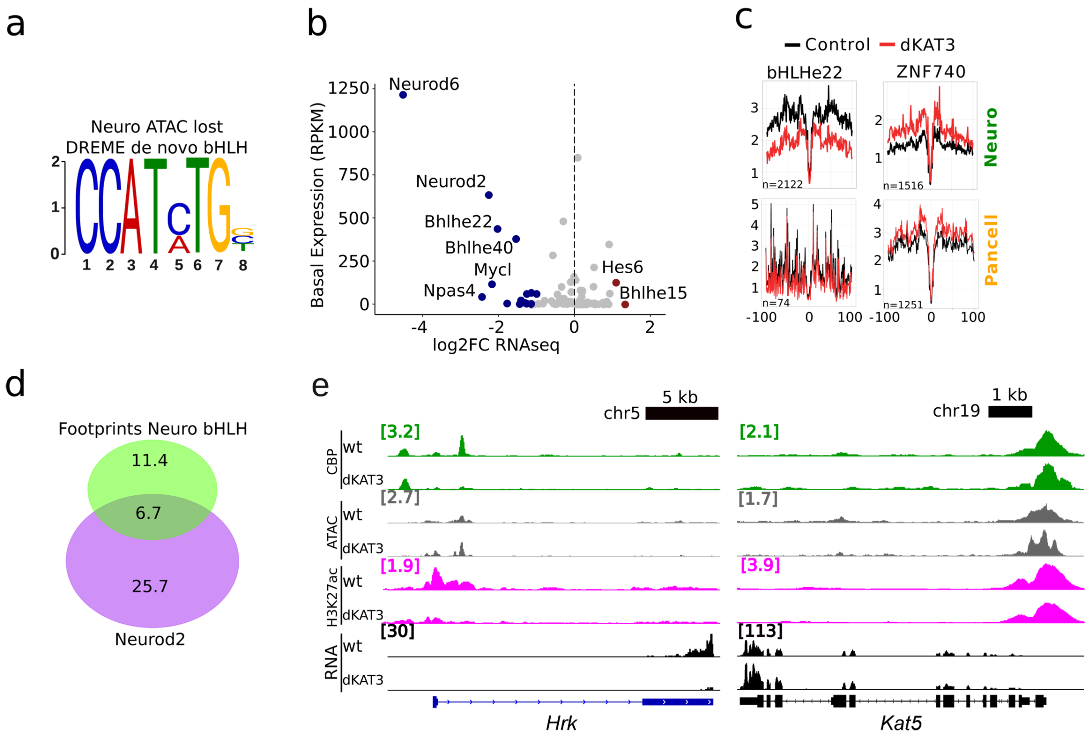


**Supplementary Figure 12 related to Figure 5. CBP and p300 are recruited by bHLH TFs.** **a.** Motif found by a motif enrichment analysis algorithm in the regions with a decreased ATAC-seq signal in dKAT3-ifKO. These regions show a signature indistinguishable from the canonical bHLH TFs binding motif. **b.** Plot showing the expression level and change in expression of bHLHs in dKAT3-ifKO hippocampus versus control mice. Every dot is a single bHLH TF expressed in the hippocampus. Blue – downregulated, red – upregulated, grey – unchanged gene expression. **c.** Left: Footprint of bHLHe22, another bHLH TF expressed in mature excitatory neurons. Right: Footprint of ZNF740, an example of TF detected in both neuronal and pancellular peaks. Values on the y-axis correspond to normalized Tn5 insertions. Values on the x-axis describe the position from the peak in bp. **d.** Overlap between Neurod2 ChIP-seq peaks and bHLH footprints found in ATAC-seq neuro-peaks. Numbers are expressed in thousands. **e.** Representative snapshots of RNA-seq, ATAC-seq, and CBP and H3K27ac ChIP profiles at the gene *Hrk* (left), which encodes a positive regulator of neuronal death strongly downregulated in dKAT3-ifKOs and belong to the category of neuronal-specific genes defined in **Figure 4**. For comparison, we also present the profiles of *Kat5*, which encodes a ubiquitous KAT that is not downregulated in dKAT3-ifKOs and was classified as pancellular gene.

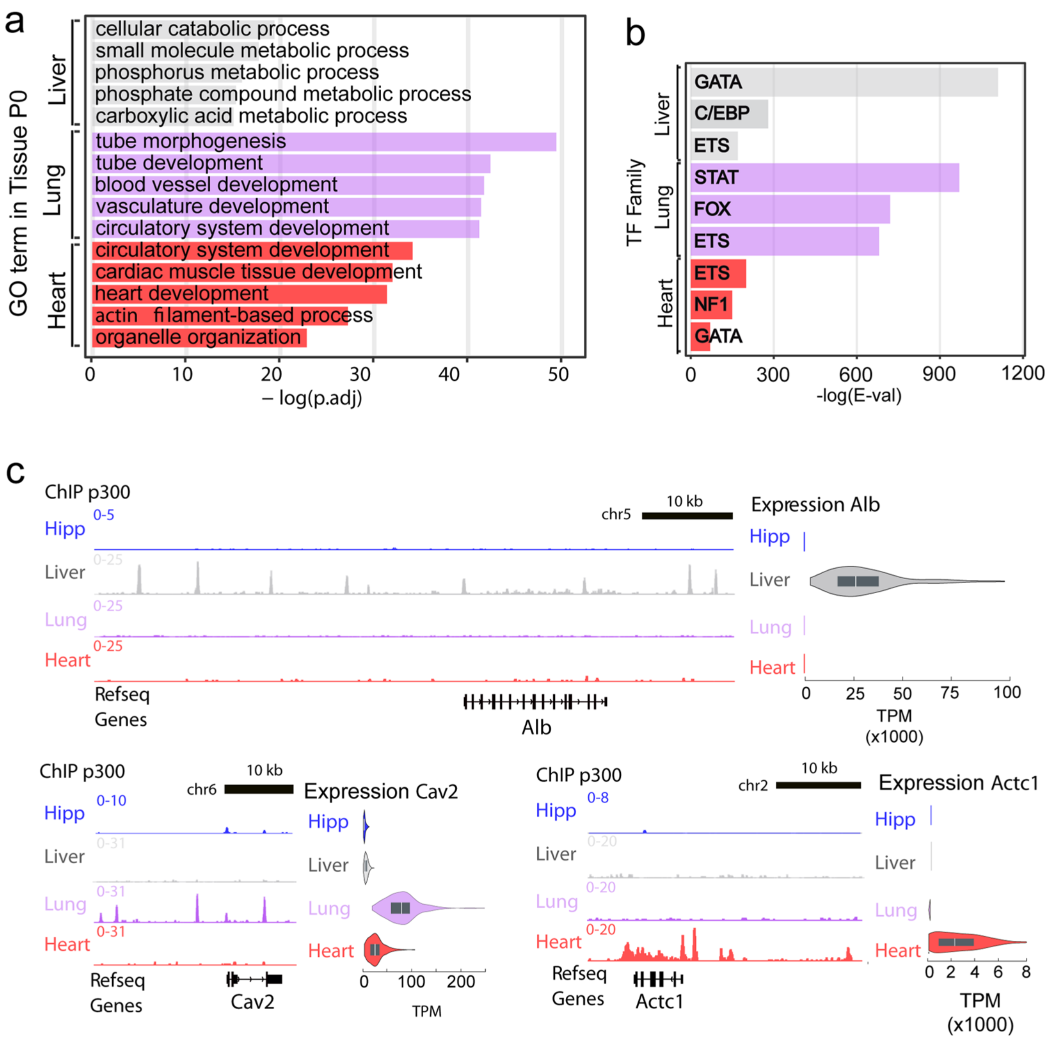


**Supplementary Figure 13 related to Figure 6. CBP and p300 also maintain other cellular type fates. a.** Top five most enriched categories identified by GO analysis on the gene sets associated with p300 binding in liver, lung and heart of P0 mice. Categories correspond to expected functions for each organ. **b.** TFBS analysis of p300-bound putative enhancers (intergenic and intronic peaks) in chromatin of liver, lung and heart tissue from P0 mice. Motif family names are taken from the most prominent results provided by the MEME-ChIP algorithm. In the case of lung and heart we detected large enrichments for specific TFs but with no clear foremost candidate as in hippocampus and liver, likely reflecting the greater cellular heterogeneity of these tissues. **c.** Representative snapshots of p300 ChIP-seq in hippocampus (this study), and liver, lung and heart (ENCODE) at three representative tissue-specific genes. Gene expression in Transcripts per Million reads (TPM) for each tissue was obtained from the Genotype-Tissue Expression (GTEx) project (www.gtexportal.org) and it is shown as violin and box plots adjacent to each track. *Alb*, *Cav2* and *Actc1* encode for Albumin, Caveolin 2 and Actin alpha cardiac muscle 1, respectively.

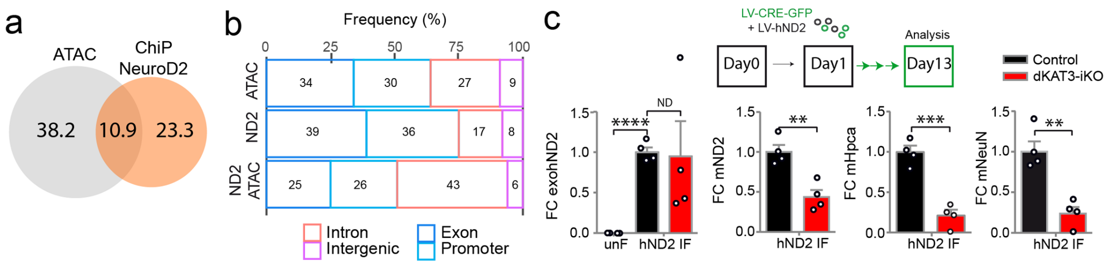


**Supplementary Figure 14 related to Figure 8. NeuroD2 overexpression does not rescue neuron-specific transcriptional deficits. a.** Overlap between NeuroD2 peaks [34] and ATAC-seq peaks in the control chromatin. **b.** Gene feature distribution of ATAC-seq enriched regions, NeuroD2 (ND2) ChIP-seq peaks and overlapping regions. **c.** RT-PCR quantifications of neuronal gene markers after LV-driven overexpression of a human version of bHLH transcription factor NeuroD2 (ND2 – *Neurod2*, NeuN – *Rbfox3*). The primers designed for ND2 can distinguish between the human (hND2) and mouse (mND2) mRNAs (n = 4 in both groups). Data are presented as mean values ± SEM. Two-tailed t-test: ****: $p < 0.0001$, ***: $p < 0.001$, **: $p < 0.01$. Source data for graphs in panel c are provided as a Source Data file.

**Supplementary Table 1 related to Figure 1. SHIRPA screen in single and double KAT3 ifKOs**

| | Test | CBP -ifKO | p300 -ifKO | dKAT3 -ifKO | CreERT2:: Crebbp$^{f/+}$:: Ep300$^{f/f}$ | CreERT2:: Crebbp$^{f/f}$:: Ep300$^{f/+}$ |
|---|---|---|---|---|---|---|
| Spontaneous activity | MW | ns | ns | ns/0.04 | ns/ns | ns/ns |
| Transfer arousal | MW | ns | ns | ns/ns | ns/ns | ns/ns |
| Fear | F | ns | ns | ns/ns | ns/ns | ns/ns |
| Piloerection | F | ns | ns | ns/ns | ns/ns | ns/ns |
| Body position | MW | ns | ns | ns/0.01 | ns/ns | ns/ns |
| Positional passivity | MW | ns | ns | ns/ns | ns/ns | ns/ns |
| Gait | MW | ns | ns | ns/ns | ns/ns | ns/ns |
| Pelvic elevation | MW | ns | ns | ns/ns | ns/ns | ns/ns |
| Tail elevation | MW | ns | ns | ns/ns | ns/ns | ns/ns |
| Visual placing | MW | ns | ns | ns/ns | ns/ns | ns/ns |
| Grip strength | MW | ns | ns | ns/0.03 | ns/ns | ns/ns |
| Wire maneuver | MW | ns | ns | ns/ns | ns/ns | ns/ns |
| Touch escape | MW | ns | ns | ns/ns | ns/ns | ns/ns |
| Trunk curl | F | ns | ns | ns/ns | ns/ns | ns/ns |
| Limb grasping | F | ns | ns | ns/ns | ns/ns | ns/ns |
| Provoked biting | F | ns | ns | ns/ns | ns/ns | ns/ns |
| Irritability | F | ns | ns | ns/ns | ns/ns | ns/ns |
| Aggression | F | ns | ns | ns/ns | ns/ns | ns/ns |
| Vocalization | F | ns | ns | ns/ns | ns/ns | ns/ns |
| Righting reflex | MW | ns | ns | ns/ns | ns/ns | ns/ns |
| Contact righting reflex | F | ns | ns | ns/ns | ns/ns | ns/ns |
| Negative geotaxis | MW | ns | ns | ns/0.03 | ns/ns | ns/ns |
| Pinna reflex | MW | ns | ns | ns/0.02 | ns/ns | ns/ns |
| Corneal reflex | MW | ns | ns | ns/ns | ns/ns | ns/ns |
| Palpebral closure | MW | ns | ns | ns/ns | ns/ns | ns/ns |
| Toe pinch | MW | ns | ns | ns/ns | ns/ns | ns/ns |
| Heart rate | MW | ns | ns | ns/ns | ns/ns | ns/ns |
| Respiration rate | MW | ns | ns | ns/ns | ns/ns | ns/ns |
| Tremor | MW | ns | ns | ns/ns | ns/ns | ns/ns |
| Barbering | MW | ns | ns | ns/ns | ns/ns | ns/ns |
| Body tone | MW | ns | ns | ns/ns | ns/ns | ns/ns |
| Abdominal tone | MW | ns | ns | ns/ns | ns/ns | ns/ns |
| Limb tone | MW | ns | ns | ns/ns | ns/ns | ns/ns |
| Skin color | MW | ns | ns | ns/ns | ns/ns | ns/ns |
| Head morphology | F | ns | ns | ns/ns | ns/ns | ns/ns |
| Lacrimation | MW | ns | ns | ns/ns | ns/ns | ns/ns |
| Salivation | MW | ns | ns | ns/ns | ns/ns | ns/ns |

Table shows results of an adapted SHIRPA test. CBP-ifKO, p300-ifKO, CreERT2::Crebbp$^{f/+}$::Ep300$^{f/f}$ and CreERT2::Crebbp$^{f/f}$::Ep300$^{f/+}$ do not show any neurological phenotype. dKAT3-ifKO were affected only after tamoxifen (TMX) treatment. In the dKAT3-ifKO column, the results before and after TMX are separated by a "/" sign. Numbers represent p-value of comparison. Statistical test used for the comparison is indicated in the column "Test": F – Two-sided Fisher Exact test, MW – Two-sided Mann-Whitney U test. ns = non-significant. Source data are provided as a Source Data file.

**Supplementary Table 2 related to Methods. Oligonucleotide pairs used in this study:**

| Target | Type | Forward | Reverse |
|---|---|---|---|
| Gapdh | RT-qPCR primer pair for detection of mRNA | CTTCACCACCATGGAGAAGGC | CATGGACTGTGGTCATGAGCC |
| CBP | RT-qPCR primer pair for detection of mRNA | TCAGCTCTTCCAACTTCCTTGG | AAGGAGGCGCTGCTGTAGGTAT |
| p300 | RT-qPCR primer pair for detection of mRNA | AAAAGACCGACGGATGGAAAA | TTCTCGGCTAGGAGGTGATAGT |
| NeuN | RT-qPCR primer pair for detection of mRNA | GCAGTCGCGGTTGGAGTAGT | CGTTAAAAATGATCTCCACGTCTAAAAT |
| Gfap | RT-qPCR primer pair for detection of mRNA | GGACAACTTTGCACAGGACCTC | TCCAAATCCACACGAGCCA |
| Hpca | RT-qPCR primer pair for detection of mRNA | CTACATCAGCCGGGAGGAGAT | ATCTTGTAAATGGCCTGCACAA |
| Grik3 | RT-qPCR primer pair for detection of mRNA | GTACGGTGCTGTCAAGGACG | GGCCCACATCTTCTCAAAGGT |
| Gria1 | RT-qPCR primer pair for detection of mRNA | TGGAAGCAAGGACTCCGGAAGT | AACTCGATTAAGGCAACCAGCATG |
| Kcnq2 | RT-qPCR primer pair for detection of mRNA | CGTTCATCTACCACGCCTACG | GCACAAGGCAGGAGAAGACTAAAA |
| Kcnq5 | RT-qPCR primer pair for detection of mRNA | TGGCTTCAAGTTGCCTCTTAATTC | CAAAGACAACGATCATCACGAAC |
| mNeuroD2 | RT-qPCR primer pair for detection of mRNA | GAGATCCCTGAACCCACGTT | TCATCTTGCGTTTCTTCGGC |
| hNeuroD2 | RT-qPCR primer pair for detection of mRNA | CTCGCCCGACCACGA | GCGCCGAGTAGTGCATAGA |
| Ppia | RT-qPCR primer pair for detection of mRNA | AAGACTGAATGGCTGGATGG | TCGGAAATGGTGATCTTCTTG |
| Pgk1 | RT-qPCR primer pair for detection of mRNA | GTCGTGATGAGGGTGGACTT | AAGGACAACGGACTTGGCTC |
| Rpl23 | RT-qPCR primer pair for detection of mRNA | ATGTCAAGGAGCTGGAGGTG | GGGATTGGTGACTCTGATGG |
| Fos Pr | ChIP-qPCR primer pair for *Fos* promoter | CGCCCAGTGACGTAGGAAGT | GCAGTCGCGGTTGGAGTAGT |
| Bdnf Pr 3 | ChIP-qPCR primer pair for *BDNF* promoter 3 | GACCAATCGAAGCTCAACCG | GGCACTGGGGTCAGACATTA |
| Intergenic | ChIP-qPCR primer pair for region ~75 kb upstream of *Bdnf* | CTACCGAGTGTTGATTGCCGT | TGATGCAAGTGTCAAGCTCAATG |
| Control gRNA | gRNA cloning (targeted to Hpca) | GCTGGCCCTGATTTCGGGC | GGCCCGAAATCAGGGCCAG |
| Neurod2 gRNA A | gRNA cloning | GGGGTACCAGCCTCTATGCC | GGCATAGAGGCTGGTACCCC |
| Neurod2 gRNA B | gRNA cloning | CCCCATTGTTCCCATGTGGG | CCCACATGGGAACAATGGGG |
| Neurod2 gRNA C | gRNA cloning | GAGATGCCACACTCGCTCCG | CGGAGCGAGTGTGGCATCTC |
| Neurod2 gRNA D | gRNA cloning | GTGGTGGGGGGGCGCTGCTT | AAGCAGCGCCCCCCCACCAC |
