# Supplementary material for: KAT3-dependent acetylation of cell type-specific genes maintains neuronal identity in the adult mouse brain
Source: Nat Commun. 2020 May 22;11:2588. doi: 10.1038/s41467-020-16246-0 (PMC7244750; doi:10.1038/s41467-020-16246-0)
Supplement: Supplementary file 3 — Description of Additional Supplementary Files [file 41467_2020_16246_MOESM3_ESM.docx]

Description for Additional Supplementary Files

**Supplementary Movie 1 related to Figure 1 (MP4 file).** Rapid emergence of neurological phenotypes in dKAT3-ifKOs.

**Supplementary Data 1 related to Figure 2.** 3 sheets: (i) RNA-seq sample description; (ii) Result of differential expression analyses for all genes; (iii) Differentially expressed genes in the dKAT3-ifKOs.

**Supplementary Data 2 related to Figure 3.** 14 sheets: (i) Top significant changes retrieved in the differential expression screen for population-enriched genes; (ii) Top enriched genes (most upregulated genes) retrieved in the differential expression screen for population-enriched genes; (iii to xiv) Results of differential expression screen for population-enriched genes in each of the 12 clusters.

**Supplementary Data 3 related to Figure 4**. 5 sheets: (i) CBP and P300 ChIP-seq sample description; (ii) BED file for all KAT3 peaks; (iii) Differential KAT3 binding in dKAT3-ifKO vs control for neuronal KAT3 peaks; (iv) Differential KAT3 binding in dKAT3-ifKO vs control for pancellular KAT3 peaks; (v) Differential KAT3 binding in dKAT3-ifKO vs control for non-neuronal KAT3 peaks.

**Supplementary Data 4 related to Figure 4.** 3 sheets: (i) GO Enrichment analysis for genes associated with neuronal KAT3 peaks; (ii) GO Enrichment analysis for genes associated with pancellular KAT3 peaks; (iii) GO Enrichment analysis for genes associated with non-neuronal KAT3 peaks. Consejo Superior de Investigaciones Científicas Universidad Miguel Hernández Instituto de Neurociencias Angel Barco abarco@umh.es www.ina.umh.es Tel: +34 965-919232 Fax: +34 965 919492 Av Ramón y Cajal s/n CAMPUS DE SAN JUAN 03550 SANT JOAN D’ALACANT– ESPAÑA

**Supplementary Data 5 related to Figure 4.** 4 sheets: (i) ATAC-seq sample description; (ii) Global Differential Accessibility in dKAT3-ifKO vs control for KAT3 peaks; (iii) Differential Accessibility in dKAT3-ifKO vs control for neuronal KAT3 peaks; (iv) Differential Accessibility in dKAT3-ifKO vs control for pancellular KAT3 peaks.

**Supplementary Data 6 related to Figure 5.** 5 sheets: (i) H3K27ac ChIP-seq sample description; (ii) Differential enrichment for H3K27ac in all peaks; (iii) Differential H3K27ac in dKAT3-ifKO vs control for neuronal peaks; (iv) Differential H3K27ac in dKAT3-ifKO vs control for pancelular peaks; (v) Differential H3K27ac in dKAT3-ifKO vs control for non-neuronal peaks.

**Supplementary Data 7 related to Figure 5.** 3 sheets: (i) list of retrieved enhancers (Enh) and super enhancers (SEnh); (ii) list of neuronal specific enhancers (Enh) and super enhancers (SEnh) detected in controls; (iii) list of neuronal specific enhancers (Enh) and super enhancers (SEnh) lost in dKAT3-ifKO.
