# Supplementary material for: KAT3-dependent acetylation of cell type-specific genes maintains neuronal identity in the adult mouse brain
Source: Nat Commun. 2020 May 22;11:2588. doi: 10.1038/s41467-020-16246-0 (PMC7244750; doi:10.1038/s41467-020-16246-0)
Supplement: Supplementary file 12 — Reporting Summary [file 41467_2020_16246_MOESM12_ESM.pdf]

nature research

Corresponding author(s): Angel Barco

Last updated by author(s): Apr 8, 2020

# Reporting Summary

Nature Research wishes to improve the reproducibility of the work that we publish. This form provides structure for consistency and transparency in reporting. For further information on Nature Research policies, see Authors & Referees and the Editorial Policy Checklist .

## Statistics

For all statistical analyses, confirm that the following items are present in the figure legend, table legend, main text, or Methods section.

| n/a | Confirmed | |
|---|---|---|
| ☐ | ☒ | The exact sample size (*n*) for each experimental group/condition, given as a discrete number and unit of measurement |
| ☐ | ☒ | A statement on whether measurements were taken from distinct samples or whether the same sample was measured repeatedly |
| ☐ | ☒ | The statistical test(s) used AND whether they are one- or two-sided<br>*Only common tests should be described solely by name; describe more complex techniques in the Methods section.* |
| ☐ | ☒ | A description of all covariates tested |
| ☐ | ☒ | A description of any assumptions or corrections, such as tests of normality and adjustment for multiple comparisons |
| ☐ | ☒ | A full description of the statistical parameters including central tendency (e.g. means) or other basic estimates (e.g. regression coefficient) AND variation (e.g. standard deviation) or associated estimates of uncertainty (e.g. confidence intervals) |
| ☐ | ☒ | For null hypothesis testing, the test statistic (e.g. *F*, *t*, *r*) with confidence intervals, effect sizes, degrees of freedom and *P* value noted<br>*Give P values as exact values whenever suitable.* |
| ☒ | ☐ | For Bayesian analysis, information on the choice of priors and Markov chain Monte Carlo settings |
| ☐ | ☒ | For hierarchical and complex designs, identification of the appropriate level for tests and full reporting of outcomes |
| ☐ | ☒ | Estimates of effect sizes (e.g. Cohen's *d*, Pearson's *r*), indicating how they were calculated |

*Our web collection on statistics for biologists contains articles on many of the points above.*

## Software and code

Policy information about availability of computer code

| | |
|---|---|
| Data collection | Equipment software of Applied Biosystems 7300 real-time PCR unit, FACS Aria III, Illumina HiSeq2500, Leica stereo microscope, Olympus confocal microscope and JEOL JEM 1400 transmission electron microscope |
| Data analysis | General sotware: Microsoft Excel 14.7.3; Adobe Photoshop and Adobe Illustrator CS6.<br><br>Statistics: R Studio v3.5.1; GraphPad Prism v8.0.1; MATLAB R2016b.<br><br>Image analysis: Fiji – ImageJ 2.0.0-rc-68; Neurolucida (MBF)<br><br>Electrophysiology analysis: Spike2 v6; MC_Stimulus v3.4; ICAofLFPs v1.02.<br><br>Genomic analysis: Cutadapt v1.18; Samtools v1.9; Bedtools v2.27.1; DeepTools v3.2.0; IGV v2.5.0; DESeq2 v1.10.0; HISAT2 v2.1.0; HTseq v0.11.1; GOstats v2.44.0; Picardtools v2.18.21; Bowtie2 v2.3.4.2; MACS2 v2.1.1; BETA v1.0.7; DiffBind v2.6.6; ChIPpeakAnno v3.20.1; HINT v0.12.3; MEME-suite v4.12.0; ChIPseeker v1.22.1; FastQC v0.11.9; Circlice v0.4.8; Cell Ranger v2.2.0; Seurat v2.3.4; Monocle2 v.2.8.0.<br><br>We used the following publicly available webtools: GEne SeT AnaLysis Toolkit (http://www.webgestalt.org/) and MEME-suite (MEME-suite.org/). The p300 ChIP-seq data for heart (ENCSR777VNA), liver (ENCSR765RPR) and lung (ENCSR527DME) of C57BL/6 P0 mice was obtained from ENCODE (https://genome.ucsc.edu/ENCODE/downloadsMouse.html). |

For manuscripts utilizing custom algorithms or software that are central to the research but not yet described in published literature, software must be made available to editors/reviewers. We strongly encourage code deposition in a community repository (e.g. GitHub). See the Nature Research guidelines for submitting code & software for further information.

## Data

Policy information about availability of data

All manuscripts must include a data availability statement. This statement should provide the following information, where applicable:

- Accession codes, unique identifiers, or web links for publicly available datasets
- A list of figures that have associated raw data
- A description of any restrictions on data availability

Data can be accessed at the GEO repository using the accession number GSE133018

# Field-specific reporting

Please select the one below that is the best fit for your research. If you are not sure, read the appropriate sections before making your selection.

☒ Life sciences ☐ Behavioural & social sciences ☐ Ecological, evolutionary & environmental sciences

For a reference copy of the document with all sections, see nature.com/documents/nr-reporting-summary-flat.pdf

# Life sciences study design

All studies must disclose on these points even when the disclosure is negative.

| | |
|---|---|
| Sample size | Sample size was determined according to data variance and correlation between biological replicates and distance to control condition. Samples sizes were sufficient according to statistical methods, confidence intervals and p-values. Each analysis was done in accordance to the sample size, sequencing depth and conditions. |
| Data exclusions | No data was excluded from the analyses |
| Replication | All replication attempts of the experiments were successful.<br>The measures applied to evaluate replicates were correlation among samples, variance, Euclidean classification, principal component analysis (PCA) and statistical tests that revealed non significant differences within the group. In the analyses in which a replicate was not available, analyses were supported by the use of internal controls and the sample was normalized and compared with samples from related conditions in which replicates were available (i.e., ANOVA, DESeq2).<br>All of the histological experiments (IHC, NISSL, Golgi, TUNEL) were replicated at least twice or had at least two biological replicates. The virus unilateral injection was replicated twice. All of the molecular experiments (ChIP, mRNA), with the exception of ChIP for H3K9,14ac were replicated twice and validated by qPCR. |
| Randomization | Set of mice with the same age and sex were raised in the same conditions and randomly allocated to the different experimental groups. Researchers were blinded to group allocation during data collection. |
| Blinding | Researchers were blinded to group allocation during data collection in all the experiments in which the subjective view of the experimenter could influence the result. |

# Reporting for specific materials, systems and methods

We require information from authors about some types of materials, experimental systems and methods used in many studies. Here, indicate whether each material, system or method listed is relevant to your study. If you are not sure if a list item applies to your research, read the appropriate section before selecting a response.

### Materials & experimental systems

| n/a | Involved in the study | |
|---|---|---|
| ☐ | ☒ | Antibodies |
| ☒ | ☐ | Eukaryotic cell lines |
| ☒ | ☐ | Palaeontology |
| ☐ | ☒ | Animals and other organisms |
| ☒ | ☐ | Human research participants |
| ☒ | ☐ | Clinical data |

### Methods

| n/a | Involved in the study | |
|---|---|---|
| ☐ | ☒ | ChIP-seq |
| ☐ | ☒ | Flow cytometry |
| ☒ | ☐ | MRI-based neuroimaging |

## Antibodies

| | |
|---|---|
| Antibodies used | The following primary antibodies have been used in this study: anti-CBP, Santa Cruz sc-583 (IHC: 1:500; ChIP: 10 µg); anti-CBP, Santa Cruz sc-369 (ICC: 1:100); anti-CBP, Santa Cruz sc-7300 (IHC: 1:100; ICC: 1:100); anti-p300, Santa Cruz sc-585 (IHC: 1:100; ICC: 1:100; ChIP: 10 µg); anti-NeuroD2, Abcam ab109406 (ICC: 1:100); anti-H2Aac, 70 (IHC: 1:100); anti-H2Bac, 70 (IHC: 1:1000; ICC: 1:1000); anti-H3K9,14ac, 70 (IHC: 1:400); anti-H3K27ac, Abcam ab4729 (IHC: 1:1000; ICC: 1:1000; ChIP: 5 µg); anti- |

H3K27me3 07-449 Millipore (IHC: 1:100); anti-H3K9me3 ab8898 Abcam (IHC: 1:100); anti-H4ac, 70 (IHC: 1:100); anti-NeuN, MAB377 Millipore (IHC: 1:500; FANS: 1:500); anti-Hpca, Abcam ab24560 (IHC: 1:500; ICC: 1:500); anti-CaMKIV, BD Transduction Laboratories C28420 (IHC: 1:500); anti-Cleaved-Cas3, Cell Signalling #9661 (IHC: 1:200); anti-Fos, Synaptic Systems #226004 (IHC: 1:500); anti-mCherry/dsRed, Clontech 632496 (ICC: 1:1000); anti-GFP, Aves Labs GFP-1020 (IHC: 1:1000; ICC: 1:1000); anti-GFAP, Sigma G9269 (IHC: 1:200; ICC: 1:100); anti-H2A.Xγ, Abcam ab2893 (IHC: 1:200). Biotinylated anti-mouse (Sigma B0529, 1:500) and anti-rabbit (Sigma B8895, 1:3000) antibodies were used in the DAB staining. Fluorophore-coupled secondary antibodies were acquired from Invitrogen and used in a dilution 1:400.

Validation

The antibodies anti-H2Aac, anti-H2Bac, anti H4ac and anti-H3K9,14ac were produced in our laboratory and validated in Lopez-Atalaya et al., Nucleic Acids Res 41:8072-84.

All the commercial antibodies are validated by the manufacturer and/or scientific publications. Information is available at http://antibodyregistry.org/

# Animals and other organisms

Policy information about studies involving animals; ARRIVE guidelines recommended for reporting animal research

Laboratory animals

Mouse strains were maintained in a pure C57BL/6J background. Previously described CaMKIIα-creERT2 (Erdmann et al. 2007), Ep300f/f (Kasper et al. 2006) and Crebbpf/f (Zhang et al. 2004) mice were crossed for the purpose of obtaining double and triple conditional knockouts. Both male and female mice were used in the experiments.

CaMKIIα-creERT2-carrying mouse lines were maintained in their standard housing until they were 3-4 month old in order for them to fully develop the central nervous system. Then, the they were treated with tamoxifen (TMX) to trigger the elimination of p300, CBP or both proteins. Experiments were performed at least 30 days after the first tamoxifen administration, unless explicitly stated otherwise. Therefore, mice were 4-5 month old at the time of data collection.

In the experiments involving stereotaxic surgeries and AVV transduction, 3-month old Crebbpf/f::Ep300f/f mice were used.

Primary hippocampal and cortical cultures were prepared from Crebbpf/f::Ep300f/f or Crebbpf/f::Ep300f/f::CAG/loxP/STOP/loxP /tdTomato embryos obtained from 3-month old pregnant mothers with the same genotypes.

Astrocyte primary cultures were prepared from cortices of P1-P3 Crebbpf/f::Ep300f/f pups.

Wild animals

The study does not involve the use of wild animals

Field-collected samples

The study does not involve field-collected samples

Ethics oversight

Animals were housed according to the Spanish and European regulations and the experiments were approved by the Animal Welfare Committee at the Instituto de Neurociencias and the CSIC Ethical Committee.

Note that full information on the approval of the study protocol must also be provided in the manuscript.

# ChIP-seq

## Data deposition

☒ Confirm that both raw and final processed data have been deposited in a public database such as GEO.

☒ Confirm that you have deposited or provided access to graph files (e.g. BED files) for the called peaks.

Data access links
*May remain private before publication.*

https://www.ncbi.nlm.nih.gov/geo/query/acc.cgi?acc=GSE133018

Files in database submission

Processed files:
RNAseq_wt.bw, RNAseq_dKAT3-ifKO.bw,
ATAC_wt.bw, ATAC_dKAT3-ifKO.bw,
wt_ChIP_aCBP.bw, wt_ChIP_aP300.bw, p300-ifKO_ChIP_aCBP.bw, p300-ifKO_ChIP_aP300.bw, cbp-ifKO_ChIP_aCBP.bw, cbp-ifKO_ChIP_aP300.bw, dKAT3-ifKO_ChIP_aCBP.bw, dKAT3-ifKO_ChIP_aP300.bw,
wt_ChIP_aH3K27Ac.bw, dKAT3-ifKO_ChIP_aH3K27Ac.bw,
wt_ChIP_aH3K9,14Ac.bw, dKAT3-ifKO_ChIP_aH3K9,14Ac.bw
filtered_Gene_BC_Matrices for for wt_snRNAseq, dKAT3_15d_snRNAseq and dKAT3_30d_snRNAseq

Raw files:
RNAseq_wt_1.fastq.gz, RNAseq_wt_2.fastq.gz, RNAseq_wt_3.fastq.gz, RNAseq_dKAT3-ifKO_1.fastq.gz, RNAseq_dKAT3-ifKO_2.fastq.gz, RNAseq_dKAT3-ifKO_3.fastq.gz.

ATAC_wt_1_R1.fastq.gz, ATAC_wt_1_R2.fastq.gz, ATAC_wt_2_R1.fastq.gz, ATAC_wt_2_R2.fastq.gz, ATAC_dKAT3-ifKO_1_R1.fastq.gz, ATAC_dKAT3-ifKO_1_R2.fastq.gz, ATAC_dKAT3-ifKO_2_R1.fastq.gz, ATAC_dKAT3-ifKO_2_R2.fastq.gz.

wt_ChIP-aCBP_1.fastq.gz, wt_ChIP-aCBP_2.fastq.gz, wt_ChIP-aCBP_3.fastq.gz, wt_ChIP-aP300_1.fastq.gz, wt_ChIP-aP300_2.fastq.gz, wt_ChIP-aP300_3.fastq.gz, p300-ifKO_ChIP_aCBP_1.fastq.gz, p300-ifKO_ChIP_aCBP_2.fastq.gz, p300-ifKO_ChIP-aP300_1.fastq.gz, p300-ifKO_ChIP-aP300_2.fastq.gz, cbp-ifKO_ChIP-aCBP_1.fastq.gz, cbp-ifKO_ChIP-aCBP_2.fastq.gz, cbp-ifKO_ChIP-aP300_1.fastq.gz, cbp-ifKO_ChIP-aP300_2.fastq.gz, dKAT3-ifKO_ChIP_aCBP_1.fastq.gz, dKAT3-ifKO_ChIP_aCBP_2.fastq.gz, dKAT3-ifKO_ChIP-aP300_1.fastq.gz, dKAT3-ifKO_ChIP-aP300_2.fastq.gz, wt_ChIP_INPUT.fastq.gz.

wt_ChIP-aH3K27Ac_1.fastq.gz, wt_ChIP-aH3K27Ac_2.fastq.gz, dKAT3-ifKO_ChIP-aH3K27Ac_1.fastq.gz, dKAT3-ifKO_ChIP-aH3K27Ac_2.fastq.gz.

wt_ChIP-aH3K9,14Ac_1.fastq.gz, wt_ChIP-aH3K9,14Ac_2.fastq.gz, dKAT3-ifKO_ChIP-aH3K9,14Ac_1.fastq.gz, dKAT3-ifKO_ChIP-aH3K9,14Ac_2.fastq.gz.

snRNAseq_wt_1_R1.fastq.gz, snRNAseq_wt_1_R2.fastq.gz, snRNAseq_wt_2_R1.fastq.gz, snRNAseq_wt_2_R2.fastq.gz, snRNAseq_wt_3_R1.fastq.gz, snRNAseq_wt_3_R2.fastq.gz, snRNAseq_wt_4_R1.fastq.gz, snRNAseq_wt_4_R2.fastq.gz, snRNAseq_dKAT3_15days_1_R1.fastq.gz, snRNAseq_dKAT3_15days_1_R2.fastq.gz, snRNAseq_dKAT3_15days_2_R1.fastq.gz, snRNAseq_dKAT3_15days_2_R2.fastq.gz, snRNAseq_dKAT3_15days_3_R1.fastq.gz, snRNAseq_dKAT3_15days_3_R2.fastq.gz, snRNAseq_dKAT3_15days_4_R1.fastq.gz, snRNAseq_dKAT3_15days_4_R2.fastq.gz, snRNAseq_dKAT3_30days_1_R1.fastq.gz, snRNAseq_dKAT3_30days_1_R2.fastq.gz, snRNAseq_dKAT3_30days_2_R1.fastq.gz, snRNAseq_dKAT3_30days_2_R2.fastq.gz, snRNAseq_dKAT3_30days_3_R1.fastq.gz, snRNAseq_dKAT3_30days_3_R2.fastq.gz, snRNAseq_dKAT3_30days_4_R1.fastq.gz, snRNAseq_dKAT3_30days_4_R2.fastq.gz

Genome browser session
(e.g. UCSC)

No longer applicable

## Methodology

Replicates

The number of replicates varies among the different NGS techniques. In the case of ChIP-seq experiments, we considered at least two experimental replicates per group. RNAseq was performed in triplicates

Sequencing depth

RNA-seq and ChIP-seq samples were sequenced in Illumina HiSeq2500 in a single-end 50 bp length configuration. ATAC-seq samples were sequenced in Illumina HiSeq2500 in a paired-end 50 bp length. Single-nucleus RNA-seq samples were sequenced in paired-end 75 bp length.
Number of reads and mapping efficiency for each experiment is stated in each supplementary table: Tables S2, S4, S6 and S7.

Antibodies

anti-CBP (sc-583), anti-p300 (sc-585), anti-H3K27ac (ab4729), anti-H3K9,14ac (Lopez-Atalaya et al., NAR 41:8072-84).

Peak calling parameters

Reads were mapped with Bowtie2 to mm10 genome index. Peak calling was done with MACS2 with default parameters including the input sample.

Data quality

All reads were trimmed to discard adapter leftovers. Only reads longer than 25 bp, with mapq > 30 and mapping to nuclear chromosomes were used for the posterior analyses.
ChIP-seq and ATAC-seq were performed in duplicates. Independent Discovery Rate (IDR) was used to retrieve the most reproducible peaks between replicates. For KAT3s which are proteins that do not bind DNA directly IDR < 0.05 was used. For the more clean ATAc-seq IDR > 0.15 was chosen

Software

Samtools v1.9, Bowtie2 v2.3.4.2, MACS2 v2.1.1, DeepTools v3.2.0, Bedtools v2.27.1, IGV v2.5.0, DESeq2 v1.10.0, ChIPpeakAnno v3.20.1, ChIPseeker v1.22.1 and circlice v0.4.8

# Flow Cytometry

## Plots

Confirm that:

☒ The axis labels state the marker and fluorochrome used (e.g. CD4-FITC).

☒ The axis scales are clearly visible. Include numbers along axes only for bottom left plot of group (a 'group' is an analysis of identical markers).

☒ All plots are contour plots with outliers or pseudocolor plots.

☒ A numerical value for number of cells or percentage (with statistics) is provided.

## Methodology

Sample preparation

Mice were sacrified by cervical dislocation, hippocampi were microdissected and washed in PBS. Nuclei were extracted after tissue disruption with a dounce homogenizer. For the ATAC-seq experiment the nuclei were additionally purified by density gradient before sorting.

Instrument

FACS Aria III

Software

Equipment software for FACS Aria III

| | |
|---|---|
| Cell population abundance | The purity of the post-sorted fractions were 98-99% as determined by re-sorting of sorted nuclei and observation in a neubauer chamber. |
| Gating strategy | For the ATAC-seq experiment nuclei samples were first gated by their DAPI signal. Subsequently the singlets were preselected by their FSC-H to FSC-A ratio and SSC-A to SSC-H ratio. Finally, nuclei were sorted by their positive fluorescent signal of interest (NeuN-APC). Population boundaries were clearly separated by normal density.<br>For the snRNA-seq experiment the intact singlets were isolated by their FSC-H to FSC-A and SSC-A to SSC-H ratios. The correct sorting was confirmed a posteriori by staining with DAPI and re-sorting of a purified singlet nuclei aliquot. |

☒ Tick this box to confirm that a figure exemplifying the gating strategy is provided in the Supplementary Information.
